# Bicavitary eosinophilic effusion in a dog with coccidioidomycosis

**DOI:** 10.1111/jvim.15810

**Published:** 2020-05-24

**Authors:** Tara L. Piech, Jared A. Jaffey, Eric T. Hostnik, Mary E. White

**Affiliations:** ^1^ Department of Veterinary Biomedical Sciences, College of Veterinary Medicine Long Island University Brookville New York USA; ^2^ Department of Veterinary Medicine and Surgery, College of Veterinary Medicine Midwestern University Glendale Arizona USA; ^3^ Department of Veterinary Clinical Sciences, Veterinary Medical Center The Ohio State University Columbus Ohio USA; ^4^ Department of Pathology and Population Medicine, College of Veterinary Medicine Midwestern University Glendale Arizona USA

**Keywords:** coccidioides, cytology, fungal disease, infectious disease, Valley Fever

## Abstract

This is a case of coccidioidomycosis in a dog, examined for vomiting and labored breathing. Physical examination and thoracic and abdominal imaging revealed pleural and peritoneal effusions, both of which exhibited neutrophilic inflammation with a substantial eosinophilic component. The dog had positive IgM and IgG coccidioidomycosis titers at initial evaluation. The eosinophilic component of the inflammation was attributed to coccidioidomycosis. The dog underwent approximately 6 months of fluconazole treatment, with both effusions and clinical signs improving after 6 weeks. Three months after cessation of antifungal treatment, the dog developed a mid‐diaphyseal lytic and proliferative lesion in the left radius caused by *Coccidioides* spp. This case illustrates the importance of consideration of coccidioidomycosis when an eosinophilic cavitary effusion is present in dogs that live in or have traveled to endemic regions.

**Abbreviations**
AFAST: abdominal focused assessment with sonography for trauma
ALP: alkaline phosphatase
RI: reference interval
TFAST: thoracic focused assessment with sonography for trauma

## 1 INTRODUCTION

Coccidioidomycosis is the most common systemic fungal disease in the southwestern United States, and is caused by the dimorphic, saprophytic fungus *Coccidioides immitis* or *Coccidioides posadasii*.^1^ These organisms are endemic in dry climates of Arizona, California, Texas, New Mexico, and Central and South America.^2^ The incidence of pulmonary infection in people in endemic areas has substantially increased over the last 10 to 20 years.^3^, ^4^ In addition, there is a significant correlation between the rate of coccidioidomycosis in humans and a risk map for coccidioidomycosis in dogs in California.^5^ Thus, it is also likely an emerging infectious disease in dogs.

In people with coccidioidomycosis, peripheral eosinophilia can occur with acute pulmonary infections, with a higher degree of eosinophilia seen in more severe, disseminated, infections.^6^ An increased number of eosinophils in tissues is less common.^7^ There is an association between fungal and allergic disease in people, and fungal exposure and sensitization promote and worsen the clinical progression of allergic disease.^8^ Thus, in some cases, increased numbers of tissue eosinophils due to fungal disease might in part be related to hypersensitivity.

Eosinophilic effusions because of coccidioidomycosis occur in humans, are most commonly found in the pleural cavity, and are thought to result from direct invasion from a lung parenchymal lesion.^9^ Eosinophilic effusions are uncommon in animals, with most cases being secondary to underlying neoplasia.^10^ This report describes coccidioidomycosis‐related bicavitary eosinophilic effusion in a dog. In addition to neoplasia, coccidioidomycosis should also be considered as a differential diagnosis for eosinophilic effusions, particularly if the dog lives in or has traveled to an endemic area.

## 2 CASE DESCRIPTION

A 6‐year‐old, male castrated, Boxer dog was referred to the Midwestern University Companion Animal Clinic Emergency Service (day 1) for a 3‐day history of gagging and vomiting, and a 1‐day history of labored breathing. The dog was not current on routine vaccinations and had not recently been tested for heartworm disease. The dog had been living in the geographic area of presentation, which is a *Coccidioides* spp. endemic area, and had no history of travel outside this region. Notable physical examination abnormalities included a respiratory rate of 52 breaths/minute with a mild increase in effort on inspiration. Thoracic auscultation revealed decreased bronchovesicular sounds primarily on the right side of the thorax, with mild crackles. Pain was easily elicited upon abdominal palpation with no palpable masses noted.

Hematologic abnormalities included a moderate leukocytosis of 34.7 K/μL (reference interval [RI] 6.0‐17.0 K/μL), a moderate neutrophilia of 32.3 K/μL (RI 3.6‐12.3 K/μL) and a mild lymphopenia of 0.73 K/μL (RI 0.83‐4.91 K/μL). Peripheral eosinophil concentration was within reference interval at 0.18 K/μL (RI 0.04‐1.62 K/μL). Serum biochemical abnormalities included a decreased blood urea nitrogen of 5.0 mg/dL (RI 9.0‐29.0 mg/dL), hypoglycemia of 71 mg/dL (RI 75‐125 mg/dL), hyperphosphatemia of 5.3 mg/dL (RI 1.9‐5.0 mg/dL), increased alkaline phosphatase (ALP) of 990 U/L (RI 0‐140 U/L), hyperproteinemia of 9.8 g/dL (RI 5.5‐7.6 g/dL) characterized by hyperglobulinemia of 7.3 g/dL (RI 2.0‐3.6 g/dL), albumin of 2.5 g/dL (RI 2.5‐4.0 g/dL), and hypercholesterolemia of 424 mg/dL (RI 120‐310 mg/dL). Thoracic radiographs revealed moderate to marked pleural effusion and the canine pancreas‐specific lipase SNAP test was negative.

**FIGURE 1:**
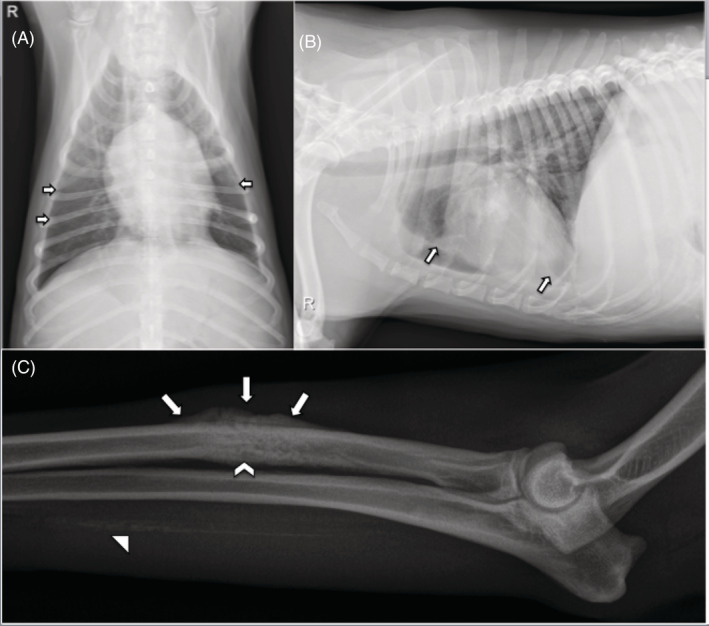
A, Ventrodorsal radiograph and, B, right lateral radiograph; soft tissue opacity causing widening of the pleural space with thin pleural fissures highlighted by white arrows. C, Irregular periosteal proliferation (white arrows) with permeative lysis (white chevron) within the mid‐diaphysis of the radius. There is also linear mineral opacity caudal to the ulna (white arrowhead). There is thickening of the soft tissue of the antebrachium

A thoracocentesis was performed at the Midwestern University Companion Animal Clinic on day 1 in which a total of 1.2 L of fluid was removed and submitted for fluid analysis and cytologic evaluation. Thoracic radiographs were performed after thoracocentesis and revealed mild residual soft tissue opacity within the pleural fissures. The pleural effusion caused rounding of the lung margins within costophrenic recesses (Figure 1A,B). Next, an abdominal ultrasonogram was performed and showed a moderate amount of echogenic fluid within the peritoneum. The hepatic veins were normal for size (Figure S1A,B). There was multifocal lobular hyperechoic mesentery with focal central, irregular hypoechoic regions (Figure S1C,D). Additional findings included an enlarged, heterogeneous hypoechoic pancreas with hyperechoic peripancreatic fat, as well as echogenic‐dependent material within the gallbladder consistent with gallbladder sludge. An abdominocentesis was performed. Based on the ultrasonographic findings and the presence of abdominal pain and bicavitary effusion, differentials included pancreatitis, gastroenteritis, systemic infectious disease, migrating foreign body, or neoplasia.

**FIGURE 2:**
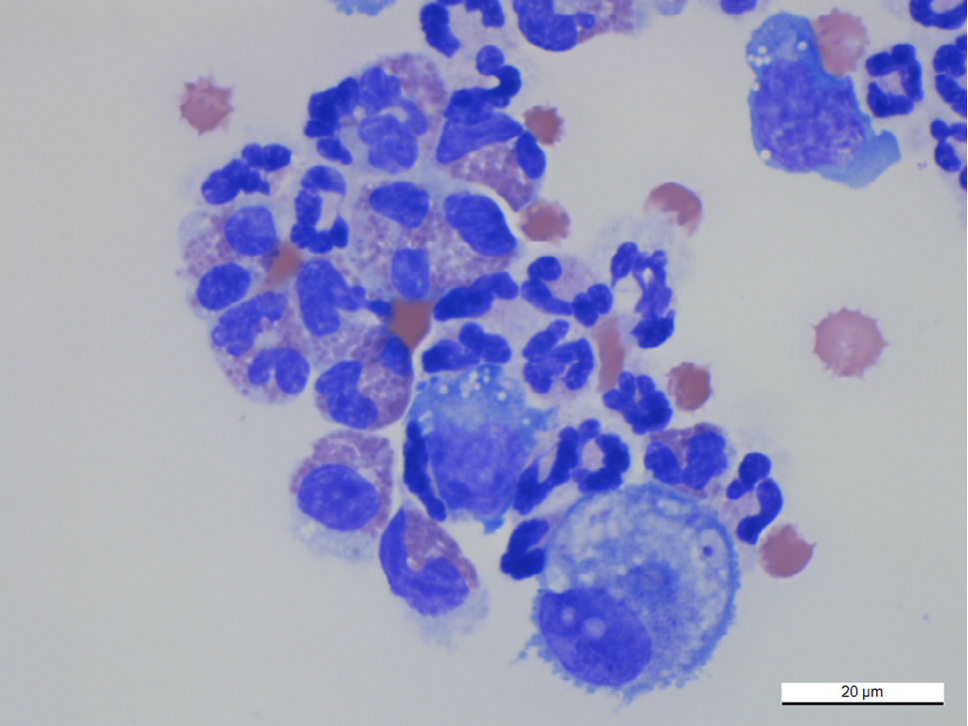
Concentrated cytospin preparation of pleural fluid. The image shows a predominance of segmented neutrophils, with lesser numbers of eosinophils and vacuolated macrophages. This image is also representative of the peritoneal fluid cytology. Wright‐Giemsa, ×50 objective

Grossly, fluid from the pleural and peritoneal cavities both appeared light yellow and clear. The pleural fluid had a total nucleated cell count of 15 510/μL and a total protein of 5.2 g/dL. The cytologic interpretation was moderate neutrophilic inflammation with an eosinophilic component, as eosinophils comprised approximately 30% of nucleated cells (Figure 2). Several vacuolated macrophages also displayed erythrophagia. The erythrophagia could have been an artifact from centrifugation, or could have indicated active hemorrhage. The latter was considered unlikely as the dog had no clinical or clinicopathologic evidence of bleeding. The peritoneal fluid had a total nucleated cell count of 52 270/μL and a total protein of 3.8 g/dL. The cytologic interpretation was marked neutrophilic inflammation with a mild eosinophilic component, as eosinophils comprised approximately 6% of nucleated cells. There were no infectious organisms or neoplastic cells seen in either fluid sample; however, neither possibility could be excluded, and investigation for underlying neoplasia or infectious disease was recommended. Results of a comprehensive fecal flotation, Baermann sedimentation, and direct smear examination, as well as heartworm antigen ELISA on heat‐treated serum (Antech Diagnostics, Fountain Valley, California) and testing for heartworm disease, Lyme disease, *Ehrlichia* spp. and *Anaplasma* spp. (SNAP 4Dx Plus Test, IDEXX Laboratories, Inc, Westbrook, Maine), were negative. The dog had positive IgM and IgG titers (IgG 1 : 32), which returned on day 8. Medical management at the time of discharge (day 1) included fluconazole (5.0 mg/kg PO q12h), prednisone (1.0 mg/kg PO q24h), maropitant (2.0 mg/kg PO q24h × 2 days), and omeprazole (1.0 mg/kg PO q12h).

The dog was presented for evaluation 2 weeks later (day 15). The dog had developed clinical signs associated with Cushing's Syndrome (polyphagia, polyuria, polydipsia, panting, and muscle loss), but had increased activity and normal respiratory rate and effort. Physical examination revealed normal bronchovesicular sounds in all lung quadrants. Thoracic and abdominal focused assessment with sonography for trauma (TFAST)/(AFAST) revealed a scant volume of pleural effusion and an absence of peritoneal effusion. The prednisone dosage (0.75 mg/kg PO q24h) was decreased and the fluconazole dosage remained unchanged. A recheck examination was recommended in 2 weeks (day 29), but the owner did not comply with this recommendation. The dog was evaluated on day 44 at which time the only abnormality on physical examination was moderate muscle wasting. Both TFAST and AFAST revealed complete resolution of pleural and peritoneal effusions. A CBC, serum biochemical profile, and urinalysis were performed and did not reveal any clinically important abnormalities. Coccidioidomycosis IgG titer decreased from 1 : 32 to 1 : 16. The prednisone dosage (0.27 mg/kg PO q24 × 10 days, then every other day × 10 days) was decreased and subsequently discontinued 20 days later while treatment with fluconazole remained unchanged.

**FIGURE 3:**
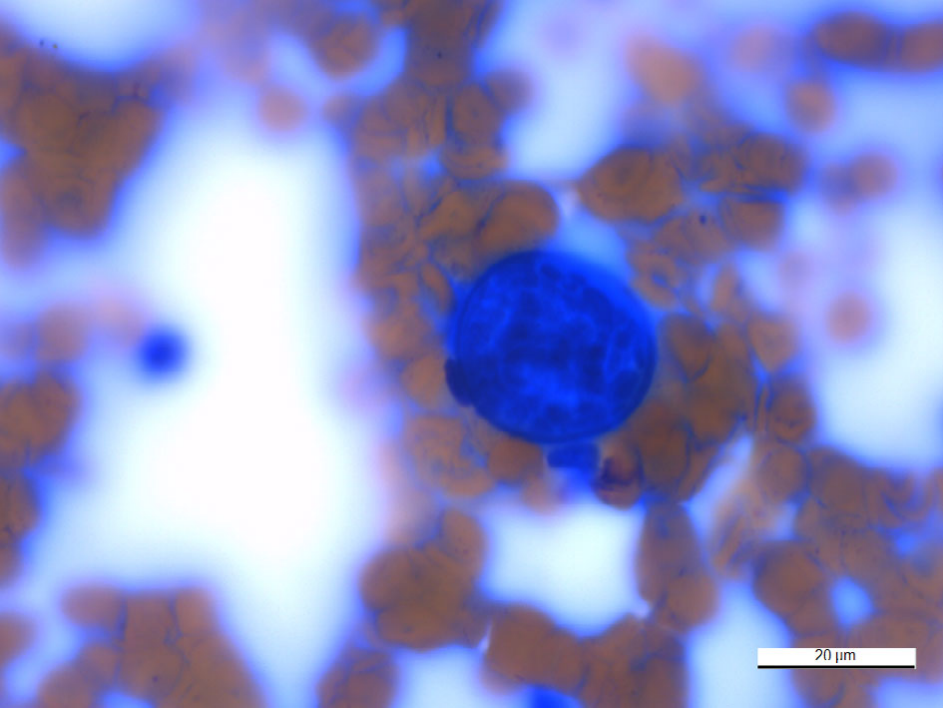
Cytology of the dog's lytic and proliferative left radial lesion. The image shows a large, approximately 70 μm, extracellular basophilic fungal spherule containing a thick, double‐contoured wall and 2 to 5 μm internal endospores, morphologically consistent with *Coccidioides* spp. Wright‐Giemsa, ×100 objective

Four months later (day 173), both IgM and IgG antibodies were negative. Fluconazole treatment was discontinued 30 days after receiving these negative titer results. Approximately 3 months after discontinuation of fluconazole (day 283), the dog was presented for evaluation of a 4‐day history of nonweight bearing lameness of the left forelimb. Physical examination revealed a small, firm mass on the left cranial antebrachium that elicited a pain response with palpation. Radiographs of the left forelimb showed focal irregular periosteal proliferation on the cranial cortical margin of the mid‐diaphyseal radius with underlying permeative lysis of the medullary bone, which was aspirated (Figure 1C). Cytologic evaluation revealed the presence of intralesional fungal spherules morphologically consistent with *Coccidioides* spp. (Figure 3). Coccidioidomycosis titers (ie, IgM and IgG) were both positive (1 : 32 IgG). Medical management at that time included carprofen (2.3 mg/kg PO q12h), tramadol (4.6 mg/kg PO q8h), and fluconazole (6.9 mg/kg PO q12h). At the time of this writing (day 346), all clinical signs associated with coccidioidomycosis have resolved and medical management consisted of only oral administration of fluconazole.

## 3 DISCUSSION

This is a report describing bicavitary eosinophilic effusion associated with coccidioidomycosis infection in a dog. Eosinophilic effusions, particularly bicavitary, are uncommon in animals, with approximately 43% (6/14) of pleural and peritoneal eosinophilic effusions in dogs and cats associated with malignancy.^10^ Associated neoplastic diseases include T‐cell lymphoma, B‐cell lymphoma, visceral mast cell neoplasia or systemic mastocytosis, and hemangiosarcoma.^10^, ^11^, ^12^, ^13^, ^14^, ^15^, ^16^, ^17^ In both dogs and cats, certain infectious agents, such as *Dirofilaria immitis*, peritoneal cestodiasis, sarcocystosis, and lungworms, can cause an eosinophilic effusion.^11^, ^18^, ^19^, ^20^ Other causes of eosinophilic effusions in dogs and cats include hypersensitivity, congestive heart failure, pneumothorax, interstitial pneumonia, disseminated eosinophilic granulomatosis, and Dacron implants (Dacron arterial conduit: USCI Division, C.R. Bard Inc, Billerica, Massachusetts), and can present as a single cavitary or as bicavitary effusion.^10^, ^11^, ^21^ Other documented causes of bicavitary effusion in animals include disseminated carcinomatosis, liver lobe torsion, and visceral mast cell neoplasia.^16^, ^22^, ^23^

Compared to people, cases of coccidioidomycosis in dogs are relatively underreported. In addition, the clinical course of the disease is also different between species, with dogs developing more subclinical, chronic infections.^24^ Although not uncommon in endemic areas, pleural effusion in dogs with coccidioidomycosis is rarely reported in the literature except in association with disseminated disease involving the pericardium.^25^ Pericardial infections in dogs are associated with widespread adhesions to the epicardium, constrictive pericardial disease, and secondary right‐sided congestive heart failure. In the case presented here, the dog had bicavitary effusion, the definitive cause of which is uncertain and could have been multifactorial. Pericarditis contributing to the effusions was considered unlikely, as TFAST did not reveal pericardial effusion, and the hepatic veins appeared normal on abdominal ultrasound, decreasing the likelihood of congestion related to cardiac disease. Importantly, pericardial fluid can be minimal or absent when constrictive scarring occurs. Thus, although cardiac disease could not be entirely ruled out, it was considered unlikely. It is possible that the effusions developed separately secondary to eosinophilic infiltration in the lungs and peritoneal cavity, or due to systemic vasculitis causing “third spacing” of fluid into the pleural and peritoneal cavities.

Eosinophilic pleural effusion associated with coccidioidomycosis occurs in people, with approximately 15% (22/146) of patients hospitalized for coccidioidomycosis infection having pleural effusion.^9^ All fluids in this study were exudates with a mean pleural eosinophil count of 10.3%, which is similar to the eosinophil percentage within this dog's peritoneal effusion.^9^ The dog's pleural effusion had a comparatively higher eosinophil percentage of 30, and this could be reflective of disease differences between humans and dogs.

Peritoneal effusions related to coccidioidomycosis infection are rare, with few cases documented in the human literature.^26^, ^27^, ^28^, ^29^, ^30^ These cases include coccidioidomycosis with gastrointestinal involvement, and in a case of a liver transplant.^27^, ^29^, ^30^ In some cases, hematogenous spread from primary pulmonary infections and ingestion of pulmonary secretions have been proposed as the underlying pathogenesis.^26^ It is possible that the dog had *Coccidioides* spp. granulomas in the abdominal viscera, particularly in light of the ultrasonographic finding of hyperechoic pancreatic mesenteric nodules that could have contributed to the development of peritoneal effusion. In addition, the markedly increased serum ALP enzyme activity, and mild hypercholesterolemia at initial presentation could lend support to mild pancreatitis possibly secondary to infection. Thus, although rare, this case illustrates that the presence of an eosinophilic effusion should prompt clinicians for investigation of possible coccidioidomycosis infection in dogs that live in or have visited endemic regions.

Although concurrent neoplasia cannot be entirely excluded from consideration in the case presented here, it was considered unlikely. The dog had complete resolution of effusions in both body cavities as well as clinical improvement after prolonged fluconazole treatment.

Relapse of clinical disease after cessation of successful antifungal treatment is documented in human medicine, with relapse in approximately 29% of 34 patients, with a mean time to relapse of 7.3 months.^31^ Similarly, relapse is also common in dogs after cessation of the recommended course of antifungal treatment, and it is unclear if resolution of infection offers lifelong immunity to the organism.^32^, ^33^ Complete recovery rates generally vary with disease severity, but an overall rate of 60% has been accepted.^32^ Thus, relapse of clinical disease in the dog presented here is not an unusual occurrence.

## CONFLICT OF INTEREST DECLARATION

Authors declare no conflict of interest.

## OFF‐LABEL ANTIMICROBIAL DECLARATION

Authors declare no off‐label use of antimicrobials.

## INSTITUTIONAL ANIMAL CARE AND USE COMMITTEE (IACUC) OR OTHER APPROVAL DECLARATION

Authors declare no IACUC or other approval was needed.

## HUMAN ETHICS APPROVAL DECLARATION

Authors declare human ethics approval was not needed for this study.

## Supporting information


**Appendix** **S1**: Supporting Information
