# Supplementary figures and images for: Bicavitary eosinophilic effusion in a dog with coccidioidomycosis
Source: J Vet Intern Med. 2020 May 24;34(4):1582–6. doi: 10.1111/jvim.15810 (PMC7379003; doi:10.1111/jvim.15810)

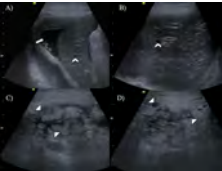

Supplement: Supplementary file 1 — Appendix S1: Supporting Information [file JVIM-34-1582-s001.pdf]
